# Maternal Dietary Patterns during Pregnancy and Congenital Heart Defects: A Case-Control Study

**DOI:** 10.3390/ijerph16162957

**Published:** 2019-08-16

**Authors:** Jiaomei Yang, Yijun Kang, Yue Cheng, Lingxia Zeng, Hong Yan, Shaonong Dang

**Affiliations:** 1 Department of Epidemiology and Health Statistics, School of Public Health, Xi’an Jiaotong University Health Science Center, Xi’an 710061, China; 2 Department of Nutrition and Food Safety, School of Public Health, Xi’an Jiaotong University Health Science Center, Xi’an 710061, China; 3 Nutrition and Food Safety Engineering Research Center of Shaanxi Province, Xi’an 710061, China; 4 Key Laboratory of Environment and Genes Related to Diseases, Xi’an Jiaotong University, Ministry of Education, Xi’an 710061, China

**Keywords:** dietary patterns, pregnancy, congenital heart defects, case-control, principal component factor analysis

## Abstract

Limited studies investigating the relationships between dietary patterns and congenital heart defects (CHDs) are available. This study aimed to explore the associations between dietary patterns and CHDs risk in Shaanxi, China. We conducted a hospital-based case-control study and included a total of 474 cases and 948 controls. Pregnant women waiting for delivery in the hospital were interviewed to report their diets during pregnancy using a validated food frequency questionnaire. Dietary patterns were derived using principal component factor analysis. Mixed logistic regression models were used to assess the associations between dietary patterns and CHDs. Pregnant women in the highest tertile of the prudent pattern had a lower risk of CHDs compared to those in the lowest tertile (OR = 0.65, 95%CI: 0.48–0.89). Pregnant women with high scores on the vegetarian pattern were at an increased risk of CHDs (medium vs. lowest tertile: OR = 1.50, 95%CI = 1.03–2.17; highest vs. lowest tertile: OR = 1.56, 95%CI = 1.13–2.15; *p*_trend_ = 0.015). Pregnant women with high scores on the dairy and egg pattern were at a reduced risk of CHDs (medium vs. lowest tertile: OR = 0.66, 95%CI = 0.49–0.90; highest vs. lowest tertile: OR = 0.60, 95%CI = 0.43–0.82; *p*_trend_ = 0.001). Maternal diet during pregnancy is an important target for intervention, and it may influence the likelihood of developing CHDs.

## 1. Introduction

Congenital heart defects (CHDs) are the most common congenital anomalies in the world, with an estimated prevalence of 9.1 per 1000 live births [1]. CHDs are the leading cause of infant morbidity and mortality from birth defects [2]. Surviving infants with CHDs may suffer from lifelong physical and mental comorbidities, posing substantial burdens on the family and society [2,3]. However, the etiology of most CHDs remains unknown. Previous studies have indicated that the occurrence of CHDs results from the interactions of genetic, environmental, lifestyle, and nutritional factors [4]. Furthermore, CHDs consist of different subtypes, including ventricular septal defects (VSD), atrial septal defects (ASD), and patent ductus arteriosus, which may have etiological heterogeneity.

The importance of optimal maternal nutrition during pregnancy has been well established [5,6]. Diet during pregnancy has been the focus of interventions to improve pregnancy outcomes [7,8]. Existing evidence suggests that some nutrients, including folate, vitamin B_12_, vitamin B_2_, and niacin are associated with CHDs [9,10,11]. In practice, different foods and nutrients are often mixed together, and the foods people consume contain thousands of nutrients. There exists a high degree of intercorrelation between nutrients and foods. Studies on individual nutrients or foods can hardly consider these complex interactions. Dietary pattern analysis has the benefit of identifying the underlying dietary characteristics of the participants, in which the consumption of foods that are eaten together can be derived [12]. Dietary pattern analysis offers a comprehensive method to examine the relationships between diets and diseases. However, there are only two published studies from the Netherlands and America investigating the associations between dietary patterns and the risk of CHDs [13,14]. Moreover, to our knowledge, no study is currently available on the relationships between dietary patterns and CHDs risk in Asia, where there are distinct dietary habits and high rates of CHDs. The present study aimed to explore the associations between dietary patterns and CHDs risk in Shaanxi, China.

## 2. Materials and Methods

### 2.1. Study Design and Participants

We conducted a hospital-based case-control study in Xi’an City, Shaanxi Province, China, from August 2014 to August 2016. We selected six tertiary comprehensive hospitals as study sites according to their qualification to conduct birth defects diagnoses and their willingness to cooperate. The six hospitals have incorporated the detailed fetal echocardiography during the 20th–24th month of gestation into their routine prenatal ultrasound screening programs, which was used as prenatal diagnosis of CHDs. Participants were recruited from among the pregnant women who were waiting for delivery in the obstetrics departments and who resided in Shaanxi Province during pregnancy. Mothers were included in the case group if their fetuses were diagnosed with isolated CHDs and had no gene disorders or chromosomal abnormalities. Mothers with fetuses diagnosed with no birth defects were included in the control group. Mothers with gestational diabetes or multiple gestations were excluded from the study because of potentially different etiologies. The controls were randomly selected each month in each hospital, and the ratio of the number of controls to cases included in the same month in the same hospital was 2:1.

Specialists from the ultrasound, pediatrics, and obstetrics departments took responsibility for the diagnoses of the cases and controls. These specialists were qualified to perform CHDs diagnoses and strictly enforced the standard diagnostic criteria. Each infant received a detailed physical examination after birth by the specialists to reconfirm the prenatal diagnoses. A telephone follow-up was also performed within one year after birth to confirm the diagnoses. All the CHDs diagnoses were ascertained by echocardiography and/or cardiac catheterization and/or surgery. Mothers with infants diagnosed with no CHDs after birth were excluded from the case group, and mothers with infants diagnosed with any birth defects after birth were excluded from the control group.

The study was conducted in accordance with the Declaration of Helsinki, and the Xi’an Jiaotong University Health Science Center approved it (approval code: 2012008). All women gave written informed consents.

### 2.2. Dietary Assessment

A 111-item semi-quantitative food frequency questionnaire (FFQ) was used to investigate maternal diets during the entire pregnancy when the eligible women were waiting for delivery in the hospital. The median time between interview completion and date of delivery was two days for both the cases and controls. Maternal dietary patterns tend to change little during pregnancy [15]; thus, we inferred that the maternal dietary pattern during the whole pregnancy was comparable with that during the critical period of cardiac development in the 3rd–8th week of gestation [16]. The FFQ was established according to a validated FFQ used for pregnant women in Shaanxi, China, as described in Reference [17]. In the validation study, the correlation coefficients for all nutrients had a mean of 0.62, with a range of 0.53 to 0.70 [17]. Participants reported consumption frequency based on eight predefined categories and recalled portion sizes with the assistance of food portion images [18,19]. Daily nutrient intakes were calculated using the Chinese Food Composition Tables [20,21].

### 2.3. Covariables

General information about the mothers was collected face-to-face using a standard questionnaire. The study covariables were classified as follows: 1) socio-demographic characteristics: maternal age (<30 years/≥30 years), residence (rural/urban), maternal occupation (farmers/others), maternal education (junior high school or below/senior high school or above), and parity (0/≥1); 2) maternal health-related factors during the first trimester: folate supplements use (yes/no), anemia (yes/no), passive smoking (yes/no), and medication use (yes/no). Mothers having no paid employment outside their homes were classified as farmers. Passive smoking was defined as being exposed to another person’s tobacco smoke for ≥15 min/d. Anemia during the first trimester was diagnosed using the criteria of hemoglobin concentration <110 g/L.

### 2.4. Statistical Analysis

The χ^2^ test or t-test was used to compare the values of categorical or continuous variables between cases and controls. The 111 dietary items were classified into 11 food groups (cereals and tubers, vegetables, fruits, red meats, white meats, eggs, dairy, legumes, nuts, snacks, and beverages) according to local eating habits, dietary guidelines [22], and the Chinese Food Composition Table [21]. Dietary patterns based on the daily intakes of the 11 food groups were derived using principal component factor analysis (PCA) with orthogonal (varimax) rotation. The Bartlett’s test of sphericity and the Kaiser–Meyer–Olkin values were adopted to evaluate the reliability of the PCA. Eigenvalues >1, the scree plot, and the interpretability of the factors were considered in determining the number of dietary patterns [23]. Food groups with absolute factor loadings >0.25 were considered the most highly related to the identified dietary pattern. The dietary pattern scores were calculated by multiplying the factor loadings with the standardized value for each food and summing across the food items [19]. The dietary pattern scores indicated the extent to which maternal diet adhered to the dietary patterns. The Mann–Whitney U test was used to compare dietary pattern scores between binary categories, and linear regression was used to calculate the P-values for linear trends in dietary pattern scores across tertiles of daily nutrient intake, in which the median for each tertile was included as a continuous variable.

In each identified dietary pattern, the mothers were grouped into three categories according to the tertiles of dietary pattern scores amongst the controls. Mixed logistic regression models were used to estimate ORs (95%CIs) for CHDs and the CHDs subtypes associated with the dietary patterns. We established three adjusted models: (1) Model 1 adjusted for total energy intake during pregnancy; (2) Model 2 adjusted for total energy intake during pregnancy and socio-demographic characteristics (maternal age, residence, occupation, education, and parity); (3) Model 3 adjusted for total energy intake during pregnancy, socio-demographic characteristics (maternal age, residence, occupation, education, and parity), and maternal health-related factors during the first trimester (folate supplements use, anemia, passive smoking, and medication use). To test for the linear trend across tertiles of the dietary pattern score, we used the median of each tertile as a continuous variable. Moreover, we conducted stratified analyses by maternal age, residence, occupation, and folate supplements use during the first trimester. We evaluated the interactions between the dietary patterns and the stratified factors by introducing cross-product terms in the regression models. All statistical analyses were performed using the Stata software (version 12.0; StataCorp, College Station, TX, USA). The tests were two-tailed with *p* < 0.05 being considered statistically significant.

## 3. Results

### 3.1. Population Characteristics

We initially recruited 560 cases with isolated CHDs and 1120 controls with no diagnosed congenital abnormality. After excluding the cases with multiple gestations and gestational diabetes, 529 cases and 1056 controls were included. Furthermore, 55 cases with chromosomal abnormalities and 108 controls with diagnosed congenital abnormalities after birth were excluded. A total of 474 cases and 948 controls who had completed the questionnaires were included in the final analysis. Table 1 shows the basic characteristics of the study sample. Case mothers were more likely to reside in rural areas, have no paid employment outside their homes, have low educational level, and be nulliparity compared to control mothers. Folate supplements use during the first trimester was more common in the controls than in the cases, while anemia, passive smoking, and medication use during the first trimester were more common in the cases than in the controls. Maternal intakes of energy and iron during pregnancy were lower among the cases than the controls. There were no differences in maternal age, neonatal gender, maternal intakes of folate, and calcium during pregnancy between the two groups. Mothers who were included in the cases and mothers who were excluded in the controls due to diagnosed congenital abnormalities after birth showed comparable general characteristics (Table S1).

### 3.2. Dietary Patterns

We identified three dietary patterns using PCA (Table 2). The Kaiser–Meyer–Olkin value was 0.830, and the *p*-value for Bartlett’s test of sphericity was <0.0001. A total of 53.39% of the variability was explained by the three dietary patterns, with the first explaining 31.22%, the second explaining 11.92%, and the third explaining 9.25%. The first pattern, which was characterized by high positive loadings on various foods, including red meats, white meats, legumes, vegetables, snacks, and dairy, was labeled as the prudent pattern. The second pattern had high positive loadings on plant-based foods, including cereals and tubers, fruits, vegetables, snacks, nuts, and legumes, and it was labeled as the vegetarian pattern. The third pattern was labeled as the dairy and egg pattern, which had high positive loadings on dairy, eggs, and nuts, and a high negative loading on beverages.

Within each dietary pattern, we looked at the distribution according to participant characteristics among the cases and controls (Table S2). In both, the cases and controls, the prudent pattern and the dairy and egg pattern scores were higher among women who resided in urban areas, had employment outside their homes, and had higher education levels, while the vegetarian pattern scores were lower among these groups. In both cases and controls, the factor score was higher among primiparous women for the prudent pattern and the dairy and egg pattern, whereas we found no difference in maternal parity for the vegetarian pattern. Pattern scores increased with increasing intakes of energy, folate, calcium, and iron during pregnancy for all the three dietary patterns.

### 3.3. Dietary Patterns and the Risk of Congenital Heart Defects

Table 3 displays the associations between the three identified dietary patterns and the risk of CHDs. In Model 1, we found that the medium and highest tertiles of the prudent pattern scores were associated with a reduced risk of total CHDs in comparison to the lowest tertile (medium vs. lowest tertile: OR = 0.63, 95%CI: 0.45–0.88; highest vs. lowest tertile: OR = 0.60, 95%CI = 0.46–0.79), and the test for trend was significant (*p*_trend_ = 0.016). In Model 2, the significant association only existed between the highest tertile of the prudent pattern score and total CHDs (OR = 0.68, 95%CI = 0.51–0.91), and the test for trend turned out to not be significant. In the fully adjusted Model 3, we observed lower risks of total CHDs (OR = 0.65, 95%CI = 0.48–0.89) and ASD (OR = 0.65, 95%CI = 0.44–0.97) for the highest tertile compared to the lowest tertile of the prudent pattern. However, we found no association between the prudent pattern and VSD and the tests for trend were not significant in the fully adjusted Model 3.

In comparison to the lowest tertile, the medium and highest tertiles of the vegetarian pattern scores were associated with a higher risk of total CHDs (medium vs. lowest tertile: OR = 1.50, 95%CI = 1.03–2.17; highest vs. lowest tertile: OR = 1.56, 95%CI = 1.13–2.15), and the test for trend was significant (*p*_trend_ = 0.015). Similarly, mothers in the medium and highest tertiles of the vegetarian pattern scores had significantly higher risks of VSD compared to those in the lowest tertile (medium vs. lowest tertile: OR = 1.97, 95%CI = 1.18–3.29; highest vs. lowest tertile: OR = 2.01, 95%CI = 1.31–3.09), and the tests for trend were significant (*p*_trend_ = 0.004). However, for the association between the vegetarian pattern and ASD, a significant result only existed when comparing the highest tertile to the lowest tertile (OR = 1.63, 95%CI = 1.08–2.46), and the test for trend was not significant.

Compared with the lowest tertile, the medium and highest tertiles of the dairy and egg pattern scores were associated with a reduced risk of total CHDs (medium vs. lowest tertile: OR = 0.66, 95%CI = 0.49–0.90; highest vs. lowest tertile: OR = 0.60, 95%CI = 0.43–0.82), and the test for trend was significant (*p*_trend_ = 0.001). Moreover, mothers in the medium and highest tertiles of the dairy and egg pattern scores had significantly lower risks of VSD (medium vs. lowest tertile: OR = 0.63, 95%CI = 0.42–0.95; highest vs. lowest tertile: OR = 0.61, 95%CI = 0.41–0.93) and ASD (medium vs. lowest tertile: OR = 0.58, 95%CI = 0.40–0.86; highest vs. lowest tertile: OR = 0.46, 95%CI = 0.30–0.70) compared to those in the lowest tertile, and the tests for trend were significant (all *p*_trend_ < 0.05).

When introducing interaction terms into the regression models, the associations of the three dietary patterns with CHDs did not meaningfully vary by maternal age, residence, occupation, or folate supplements use during the first trimester, and the tests for interactions were not significant (all *p* > 0.05).

## 4. Discussion

In this study, we identified three dietary patterns: prudent pattern, vegetarian pattern, and dairy and egg pattern. The prudent pattern and the dairy and egg pattern were found to be associated with reduced risks of CHDs and some CHDs subtypes, whereas the vegetarian pattern was associated with increased risks of CHDs and some CHDs subtypes.

To date, there have only been two studies examining the relationships between dietary patterns and CHDs risk [13,14]. One study from the Netherlands reported that the one-carbon-rich dietary pattern, characterized by high intakes of fish and seafood, was associated with a decreased risk of CHDs [13]. Another study from America found that women in the prudent dietary pattern who had high intakes of yogurt, reduced-fat milk, whole-wheat bread, fortified cereal, and fish, and low intakes of fruits and vegetables, even with folate fortification, may have lower risks of some heart defects [14]. The present study in Shaanxi, China, suggested that the prudent pattern, characterized by high intakes of red meats, white meats, legumes, vegetables, snacks, and dairy, and the dairy and egg pattern, characterized by high intakes of dairy, eggs, and nuts and low intake of beverages, were associated with lower risks of total CHDs and some CHDs subtypes. Food groups in the prudent pattern and the dairy and egg pattern of this study were comparable to those in the prudent pattern reported among pregnant women in America [14] and China [24]. The present study also suggested that the vegetarian pattern characterized by plant-based foods, including cereals and tubers, fruits, vegetables, snacks, nuts, and legumes, was associated with an increased risk of CHDs. The vegetarian pattern in the present study was similar to the pattern reported among pregnant women in Norway [25], Australia [12], and China [19,24].

There have been other studies investigating the associations between dietary patterns and birth defects, including neural tube defects [26,27], orofacial clefts [26,28], and hypospadias [29]. A study in America found that healthier dietary patterns during pregnancy, as measured by diet quality scores, reduced the risks of neural tube defects and orofacial clefts [26]. A study from the Netherlands reported that a maternal Mediterranean dietary pattern, characterized by high intakes of fruit, vegetables, vegetable oil, alcohol, fish, legumes, and cereals, and low intakes of potatoes and sweets, decreased the risk of spina bifida [27]. In the same Netherlands study, a maternal western dietary pattern, characterized by high intakes of meats, pizza, legumes, and potatoes and low intake of fruits, increased the risk of cleft lip or cleft palate [28]. A study in England found that a less health-conscious maternal dietary pattern, including a low consumption frequency of yogurt, cheese, eggs, fruit and vegetables, fish, beans and pulses, olive oil, and organic food, was associated with a higher risk of hypospadias [29]. In fact, all results from these studies suggested that dietary patterns associated with healthy diets decreased the risks of birth defects. The findings in the present study suggested that healthy diets rich in red meats, white meats, legumes, vegetables, snacks, and dairy, or rich in dairy, eggs, and nuts and low in beverages reduced the risk of CHDs, which provided more evidence of the benefits of healthy diets on birth defects. Most previous studies have reported a reduced risk of cardiovascular diseases associated with a vegetarian diet [30], which are not consistent with the results for CHDs in our study. It is noteworthy to mention that most previous studies were conducted in western populations, which have distinct dietary habits and genetic backgrounds compared to the Chinese population. Even in China, people have developed different local dietary habits in the vast territory, such as in Southern China and Northwest China. Moreover, it has been reported that different types of plant-based diets have different effects on heart diseases [31]. The discrepancy in the associations between vegetarian pattern and heart diseases and CHDs may also be due to different targeted outcomes, study designs, and nutrient assessment methods.

Maternal diet is the major intrauterine environment factor that is critical to fetal development [32]. As an important modifiable factor, maternal diet can be easily intervened with low cost and low risk. Previous studies have reported that maternal low intakes of vitamin B_12_, vitamin B_2_, and niacin increased the risk of CHDs [10,11]. Optimal iron status was reported to be critical for cardiovascular development [33]. Diets with food groups rich in these nutrients may be beneficial for development of the cardiovascular system. Pregnant women with higher scores on the prudent pattern in our study may have appropriate nutritional status as a result of higher intakes of various foods, including meats, legumes, vegetables, snacks, and dairy. Pregnant women with higher scores on the dairy and egg pattern in our study had high intakes of nutrient-dense foods, including dairy, eggs, and nuts and low intake of beverages, which may also contribute to appropriate nutritional status and thus reduced the risk of CHDs. Moreover, pregnant women with higher prudent pattern and dairy and egg pattern scores tended to be in the socio-demographically advantaged groups (in urban, having employment outside homes, and having higher education levels), as shown in Table S2. These women were more likely to have better nutritional status because they may have more opportunity to attain nutrition knowledge and pay more attention to a healthy diet. However, pregnant women with higher vegetarian pattern scores tended to be in the socio-demographically disadvantaged groups (in rural, having no employment outside homes, and having lower education levels), as shown in Table S2, which was similar to the results for vegetarian dietary pattern in the previous study conducted in the Shaanxi Province of Northwest China [19]. Pregnant women in these socio-demographically disadvantaged groups were more likely to have poor nutritional status because of the limited resources, and thus, an increased risk of CHDs. Despite the fact that folate is abundantly available in plant-based diets, and that folate supplements use may reduce the risk of CHDs [9], the amount of dietary folate intake during pregnancy was so low among the study population (219.4 mg/d in cases and 244.2 mg/d in controls as shown in Table 1). It was too low to raise the body folate level to be beneficial for the development of the cardiovascular system.

To our knowledge, this study was the first to explore the associations between dietary patterns during pregnancy and CHDs among the Chinese population, which has distinct dietary habits compared to the western population. The diagnoses of CHDs are accurate because they were confirmed by echocardiography and/or cardiac catheterization and/or surgery. We also conducted a telephone follow-up within one year after birth to eliminate the possible misclassification of cases and controls, as most birth defects were diagnosed in the first year of life. However, we should acknowledge some limitations of this study. First, due to the limited sample size, we cannot separately explore the relationships between dietary patterns and other CHDs subtypes. Further studies with large sample sizes are warranted to address this limitation. Second, affected fetuses that did not survive were not considered in the study, which may cause selection bias. Third, maternal information in pregnancy was retrospectively reported by the pregnant women waiting for delivery. Previous studies suggested that maternal diets and events during pregnancy could be recalled well after birth [34,35]. Despite this, we cannot rule out the possible limitation of recall bias, especially for the fact that women knowing their fetuses have CHDs may report events differently compared to women with normal fetuses. To minimize bias, we made efforts to help women report accurate information during the interview. For one thing, standard questionnaires and supporting materials including food portion images were adopted to collect information; for another, the survey was tested in a pilot study and interviewers were strictly trained according to the detailed guides before the formal implementation. Fourth, we collected maternal diets during the whole pregnancy rather than during the critical period of cardiac development in the 3rd–8th week of gestation, which may cause exposure misclassification. However, previous studies have suggested that maternal dietary patterns tended to change little from early to late pregnancy [15]. Finally, residual confounding cannot be ruled out despite the careful consideration of potential confounders.

## 5. Conclusions

In conclusion, our findings suggest that maternal adherence to the prudent pattern and the dairy and egg pattern during pregnancy is associated with a lower risk of CHDs, whereas maternal adherence to the vegetarian pattern during pregnancy increases the risk of CHDs. These results provide evidence to support the recommendation of healthy diets for pregnant women. Further prospective studies with large sample sizes are warranted to clarify the roles of the dietary patterns on the prevention of CHDs.

## Figures and Tables

**Table 1:** Characteristics of the study sample.

Characteristics	Case (*N* = 474)	Control (*N* = 948)	*p* *
Socio-demographic characteristics, *n* (%)			
Maternal age ≥ 30 years	159 (33.5)	324 (34.2)	0.812
Rural residence	161 (34.0)	269 (28.4)	0.030
Maternal occupation, farmers	234 (49.5)	201 (21.0)	<0.001
Maternal education, junior high school or below	195 (41.1)	183 (19.3)	<0.001
Nulliparity	274 (57.8)	761 (80.3)	<0.001
Maternal health-related factors during the first trimester, *n* (%)			
Folate supplements use	292 (61.6)	751 (79.2)	<0.001
Anemia	80 (16.9)	103 (10.9)	0.001
Passive smoking	159 (33.5)	88 (9.3)	<0.001
Medication use	197 (41.6)	288 (30.4)	<0.001
Neonatal gender, male, *n* (%)	248 (52.3)	471 (49.7)	0.348
Daily nutrient intakes during pregnancy, mean (SD)			
Energy (kcal)	1860.5 (868.0)	2044.8 (954.4)	0.001
Folate (μg)	219.4 (93.4)	244.2 (92.5)	0.146
Calcium (mg)	421.9 (187.9)	561.6 (209.5)	0.102
Iron (mg)	19.1 (8.7)	22.9 (10.9)	<0.001

* Categorical variables are compared between groups by χ^2^ test, and continuous variables are compared between groups using the *t-*test.

**Table 2:** Factor loadings for the identified dietary patterns ^1^.

Dietary Pattern	Food Groups	Factor Loading	Variance Explained, %	Cumulated Variance Explained, %
Prudent pattern			31.22	31.22
	Red meats	0.77		
	White meats	0.73		
	Legumes	0.59		
	Vegetables	0.55		
	Snacks ^2^	0.50		
	Dairy	0.28		
Vegetarian pattern			11.92	43.14
	Cereals and tubers	0.71		
	Fruits	0.66		
	Vegetables	0.56		
	Nuts	0.51		
	Snacks ^2^	0.51		
	Legumes	0.30		
Dairy and egg pattern			9.25	53.39
	Dairy	0.68		
	Eggs	0.68		
	Nuts	0.45		
	Beverages	−0.41		

^1^ The absolute factor loadings <0.25 are not shown. ^2^ Snacks include Chinese cold noodle, Chinese dessert, cake, instant noodles, dumpling, bread, rice crust, and biscuits.

**Table 3:** Associations between dietary patterns and congenital heart defects.

Dietary Patterns	Total CHDs (*N*_cases_ = 474)				VSD (*N*_cases_ = 222)	ASD (*N*_cases_ = 218)
	Cases/Controls	Model 1 OR ^1^ (95%CI)	Model 2 OR ^2^ (95%CI)	Model 3 OR ^3^ (95%CI)	Model 3 OR ^3^ (95%CI)	Model 3 OR ^3^ (95%CI)
Prudent pattern						
Tertile 1	231/316	1	1	1	1	1
Tertile 2	132/316	0.63 (0.45, 0.88)	0.79 (0.56, 1.13)	0.77 (0.53, 1.12)	0.82 (0.50, 1.32)	0.77 (0.48, 1.24)
Tertile 3	111/316	0.60 (0.46, 0.79)	0.68 (0.51, 0.90)	0.65 (0.48, 0.89)	0.75 (0.51, 1.11)	0.65 (0.44, 0.97)
*p*_trend_^4^		0.015	0.086	0.080	0.326	0.191
Vegetarian pattern						
Tertile 1	121/316	1	1	1	1	1
Tertile 2	196/316	1.96 (1.47, 2.62)	1.57 (1.17, 2.14)	1.50 (1.03, 2.17)	1.97 (1.18, 3.29)	1.31 (0.80, 2.14)
Tertile 3	157/316	2.36 (1.67, 3.34)	1.67 (1.17, 2.38)	1.56 (1.13, 2.15)	2.01 (1.31, 3.09)	1.63 (1.08, 2.46)
*p*_trend_^4^		<0.001	0.003	0.015	0.004	0.156
Dairy and egg pattern						
Tertile 1	244/316	1	1	1	1	1
Tertile 2	124/316	0.53 (0.41, 0.70)	0.63 (0.47, 0.84)	0.66 (0.49, 0.90)	0.63 (0.42, 0.95)	0.58 (0.40, 0.86)
Tertile 3	106/316	0.48 (0.36, 0.64)	0.56 (0.41, 0.75)	0.60 (0.43, 0.82)	0.61 (0.41, 0.93)	0.46 (0.30, 0.70)
*p*_trend_^4^		<0.001	<0.001	0.001	0.014	<0.001

ASD, atrial septal defects; CHDs, congenital heart defects; VSD, ventricular septal defects. ^1^ Adjusted for total energy intake during pregnancy. ^2^ Adjusted for total energy intake during pregnancy and socio-demographic characteristics (maternal age, residence, occupation, education, and parity). ^3^ Adjusted for total energy intake during pregnancy, socio-demographic characteristics (maternal age, residence, occupation, education, and parity), and maternal health-related factors during the first trimester (folate supplements use, anemia, passive smoking, and medication use). ^4^ *p* for trend across tertiles is calculated using the median for each tertile as a continuous variable.

## Supplementary Materials

The following are available online at https://www.mdpi.com/1660-4601/16/16/2957/s1, Table S1: Characteristics among mothers who were in the cases and mothers who were excluded in the controls due to diagnosed congenital abnormalities after birth; Table S2: Dietary pattern scores according to participant characteristics among the cases and controls.
