# Supplementary material for: Maternal Dietary Patterns during Pregnancy and Congenital Heart Defects: A Case-Control Study
Source: Int J Environ Res Public Health. 2019 Aug 16;16(16):2957. doi: 10.3390/ijerph16162957 (PMC6721011; doi:10.3390/ijerph16162957)
Supplement: Supplementary file 1 [file ijerph-16-02957-s001.pdf]

Table S1. Characteristics among mothers who were in the cases and mothers who were excluded in the controls due to diagnosed congenital abnormalities after birth.

Characteristics	Cases (N = 474)	Excluded Controls (N = 108)	p^*
Socio-demographic characteristics, *n (%)*			
Maternal age ≥ 30 years	159 (33.5)	38 (35.2)	0.745
Rural residence	161 (34.0)	37 (34.3)	0.954
Maternal occupation, farmers	234 (49.5)	43 (39.8)	0.073
Maternal education, junior high school or below	195 (41.1)	44 (40.7)	0.939
Nulliparity	274 (57.8)	62 (57.4)	0.940
Maternal health-related factors during the first trimester, *n (%)*			
Folate supplements use	292 (61.6)	61 (56.5)	0.325
Anemia	80 (16.9)	17 (15.7)	0.775
Passive smoking	159 (33.5)	42 (38.9)	0.292
Medication use	197 (41.6)	44 (40.7)	0.876
Neonatal gender, male, n (%)	248 (52.3)	61 (56.5)	0.434
Daily nutrient intakes during pregnancy, mean (SD)			
Energy (kcal)	1860.5 (868.0)	1839.4 (879.6)	0.775
Folate (μg)	219.4 (93.4)	215.4 (102.2)	0.699
Calcium (mg)	421.9 (187.9)	388.1 (182.4)	0.272
Iron (mg)	19.1 (8.7)	18.5 (8.9)	0.628

* Categorical variables are compared between groups by χ^2 test, and continuous variables are compared between groups by *t* test.

Table S2. Dietary pattern scores according to participant characteristics among the cases and controls.

Characteristics	Prudent pattern score[1]		Vegetarian pattern score[1]		Dairy and egg pattern score[1]	
	Case	Control	Case	Control	Case	Control
**Socio-demographic characteristics**						
**Maternal age (years)**						
< 30	- 0.51	- 0.12	- 0.06	- 0.21	- 0.56	0.18
≥ 30	- 0.51	- 0.10	- 0.04	- 0.29	- 0.46	0.15
p^2	0.600	0.780	0.659	0.268	0.622	0.322
**Residence**						
Rural	- 0.62	- 0.28	- 0.01	- 0.09	- 0.70	0.06
Urban	- 0.27	- 0.03	- 0.14	- 0.30	- 0.28	0.23
p^2	< 0.001	< 0.001	0.024	0.028	< 0.001	0.007
**Maternal occupation**						
Farmers	- 0.63	- 0.21	- 0.01	- 0.23	- 0.74	0.11
Others	- 0.41	- 0.08	- 0.16	- 0.37	- 0.34	0.19
p^2	< 0.001	0.012	0.023	0.029	< 0.001	0.014
**Maternal education**						
Junior high school or below	- 0.69	- 0.28	- 0.01	- 0.11	- 0.94	0.02
Senior high school or above	- 0.33	- 0.08	- 0.11	- 0.27	- 0.31	0.23

p^2	< 0.001	< 0.001	0.033	0.018	< 0.001	0.004
**Parity**						
0	- 0.44	- 0.11	- 0.07	- 0.26	- 0.36	0.22
≥ 1	- 0.62	- 0.22	- 0.04	- 0.23	- 0.83	0.02
p^2	0.002	0.027	0.316	0.231	< 0.001	< 0.001
**Maternal health-related factors during the first trimester**						
**Folate supplements use**						
Yes	- 0.47	- 0.11	- 0.03	- 0.29	- 0.44	0.18
No	- 0.56	- 0.14	- 0.07	- 0.20	- 0.60	0.16
p^2	0.102	0.540	0.091	0.509	0.071	0.339
**Anemia**						
Yes	- 0.51	- 0.19	- 0.04	- 0.23	- 0.63	0.11
No	- 0.50	- 0.11	- 0.05	- 0.26	- 0.46	0.19
p^2	0.287	0.606	0.175	0.283	0.215	0.153
**Passive smoking**						
Yes	- 0.55	- 0.20	- 0.03	- 0.23	- 0.58	0.14
No	- 0.45	- 0.11	- 0.07	- 0.27	- 0.48	0.19
p^2	0.416	0.244	0.615	0.325	0.196	0.302
**Medication use**						
Yes	- 0.55	- 0.13	- 0.03	- 0.20	- 0.48	0.14
No	- 0.45	- 0.15	- 0.07	- 0.29	- 0.58	0.21
p^2	0.227	0.139	0.338	0.227	0.487	0.253
**Neonatal gender**						
Male	- 0.48	- 0.16	- 0.08	- 0.18	- 0.52	0.16
Female	- 0.56	- 0.09	- 0.03	- 0.24	- 0.53	0.18
p^2	0.088	0.170	0.846	0.161	0.742	0.818
**Daily nutrient intakes during pregnancy**[3]						
**Energy**						
Tertile 1	- 0.74	- 0.43	- 0.39	- 0.81	- 0.84	- 0.04
Tertile 2	- 0.32	- 0.07	0.10	- 0.17	- 0.39	0.36
Tertile 3	0.48	0.68	0.89	0.70	0.01	0.41
p^4	< 0.001	< 0.001	< 0.001	< 0.001	< 0.001	< 0.001
**Folate**						
Tertile 1	- 0.01	- 0.08	- 0.34	- 0.18	- 0.44	- 0.21
Tertile 2	- 0.06	0.23	0.14	- 0.11	- 0.57	0.46
Tertile 3	0.60	0.90	1.03	0.81	0.06	0.41
p^4	< 0.001	< 0.001	< 0.001	< 0.001	0.017	< 0.001
**Calcium**						
Tertile 1	- 0.69	- 0.42	- 0.27	- 0.71	- 0.85	- 0.27
Tertile 2	- 0.27	- 0.13	0.10	- 0.23	0.02	0.39
Tertile 3	0.58	0.58	0.61	0.43	0.03	0.66
p^4	< 0.001	< 0.001	< 0.001	< 0.001	< 0.001	< 0.001
**Iron**						
Tertile 1	- 0.70	- 0.37	- 0.39	- 0.83	- 0.73	- 0.08
Tertile 2	- 0.38	- 0.16	0.08	- 0.19	- 0.35	0.41
Tertile 3	0.28	0.50	0.60	0.57	- 0.29	0.32
p^4	< 0.001	< 0.001	< 0.001	< 0.001	0.011	< 0.001

[1]Values are mean dietary pattern scores obtained by principal component factor analysis and the scores are not adjusted for confounders.

[2]Mann- Whitney *U* test.

[3]Participants were grouped into three categories according to the tertiles of daily nutrient intake among the controls.

[4]Test for linear trend.
